# Prompt-Contrastive Learning for Zero-Shot Relation Extraction

**DOI:** 10.3390/e28010069

**Published:** 2026-01-06

**Authors:** Xueyi Zhong, Liye Zhao, Licheng Peng, Guodong Yang, Kun Hu, Wansen Wu

**Affiliations:** 1 School of Finance, Southwestern University of Finance and Economics, Chengdu 611130, China; sammy_zhong@126.com; 2 Navy Submarine Academy, Qingdao 266000, China; zly1984qtxy@163.com (L.Z.); penglicheng1986@163.com (L.P.); guodongqtxy@163.com (G.Y.); hk_qtxy@163.com (K.H.)

**Keywords:** relation extraction, prompt learning, contrastive learning, zero-shot setting

## Abstract

Relation extraction serves as an essential task for knowledge acquisition and management, defined as determining the relation between two annotated entities from a piece of text. Over recent years, zero-shot learning has been introduced to train relation extraction models due to the expensive cost of incessantly annotating emerging relations. Current methods endeavor to transfer knowledge of seen relations into predictions of unseen relations by conducting relation extraction through different tasks. Nonetheless, the divergence in task formulations prevents relation extraction models from acquiring informative semantic representations, resulting in inferior performance. In this paper, we strive to exploit the relational knowledge contained in pre-trained language models, which may generate enlightening information for the representation of unseen relations from seen relations. To this end, we investigate a **P**rompt-**C**ontrastive learning perspective for **R**elation **E**xtraction under a zero-shot setting, namely **PCRE**. To be specific, based on leveraging semantic knowledge from pre-trained language models with prompt tuning, we augment each instance with different prompt templates to construct two views for an instance-level contrastive objective. Additionally, we devise an instance-description contrastive objective to elicit relational knowledge from relation descriptions. With joint optimization, the relation extraction model can learn how to separate relations. The experimental results show our **PCRE** method outperforms state-of-the-art baselines in zero-shot relation extraction. The further extensive analysis verifies that our proposal is robust in different datasets, the number of seen relations, and the number of training instances.

## 1. Introduction

Relation extraction (RE) is an important topic in information extraction, which identifies the semantic relation between two entities from a piece of plain text, or an instance [1,2]. RE has demonstrated significant value across diverse domains, including biomedical research [3], social media analysis [4], and especially, the financial sector, by deriving valuable insights from financial documents (e.g., news articles, earnings reports, and corporate filings) [5,6].

As a necessary step for knowledge graph automatic construction, RE plays a vital role in intelligent search and knowledge acquisition [7,8]. Over recent years, supervised relation extraction has been popular as a multi-class classification task [9,10,11], which recognizes relations for entity pairs by selecting the most appropriate relation from a pre-defined relation set. However, in practical scenarios, the training data for supervised RE models covers only a limited set of relations and cannot encompass the full spectrum of possible relation types [12]. Consequently, these models are unable to generalize to unseen relations at test time.

To alleviate the limitations in recognizing fresh relations, zero-shot relation extraction (ZSRE) has emerged as a promising approach [13,14], seeking to find unseen relations by mimicking the inference process of humans, which we formally define and contextualize in Section 2. In ZSRE, training and test relations are entirely distinct, preventing RE models from acquiring specific semantic knowledge about test relations. Consequently, ZSRE models must acquire transferable knowledge from training relations and adapt it to novel relations encountered during testing. However, the process of transferring knowledge from training to test stages poses significant challenges.

Confronted with the challenging task of ZSRE, researchers have sought solutions by formulating it as different tasks. For example, Obamuyide et al. reformulate ZSRE as a question-answering task by designing question templates for relations [13], employing meticulously designed question templates tailored to specific relations. Similarly, Levy et al. reframe ZSRE as a text entailment task, matching texts with relation descriptions [14]. Frustratingly, the diversity of existing approaches—ranging from template-based and question-answering formulations to graph-augmented architectures—leads to inconsistent input structures and learning objectives. This heterogeneity prevents models from leveraging a unified semantic space, hinders knowledge transfer across methods, and complicates fair comparison, ultimately limiting the quality of learned relation representations and degrading downstream performance.

The recent advances of pre-trained language models provide a promising way for ZSRE to learn effective representations. In a notable study, Chen et al. successfully employ the pre-trained BERT model to encode relational instances and relation descriptions, respectively, and generate zero-shot predictions by matching their representations [15]. Inspired by prompt learning [16] which bridges the gap between pre-training and fine-tuning, some of the literature resorts to concatenating relational instances with a prompt template to obtain more discriminative representations [17,18].

**Figure 1:**
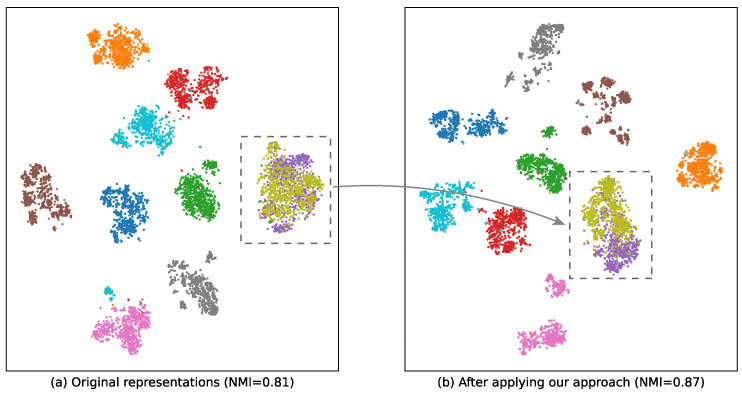
t-SNE [20] visualization (a dimensionality reduction method for visualizing high-dimensional data) of instance representations for 10 randomly selected relations from FewRel [11] (a widely used relation extraction benchmark). (**a**) Instance embeddings obtained directly from the prompt-based encoder. (**b**) Instance embeddings after applying instance-level contrastive learning. Notably, two semantically similar but distinct relations that are entangled in (**a**) become clearly separated in (**b**). The higher Normalized Mutual Information (NMI) score in (**b**) further confirms improved clustering quality.

Although recent efforts on ZSRE have achieved remarkable improvements, there are two imperfections that degrade the ability to extract fresh relations: (1) Existing ZSRE approaches [17,18] that harness a prompt-based encoder still adhere to the traditional fine-tuning paradigm. In other words, they incorporate additional classification layers for relation prediction, thereby deviating from the original intent of prompt tuning [16]. The remaining gap between pre-training and fine-tuning prevents the pre-trained language model from fully acquiring a comprehensive understanding of relational knowledge. (2) When we apply a prompt-based encoder to encoding test samples without additional supervision, the derived instance embeddings are proved to be of low quality. As shown in Figure 1a, we visualize the relation embeddings produced by a standard prompt-based encoder using t-SNE. Each point corresponds to an instance embedding, colored by its ground-truth relation label. Notably, semantically similar but distinct relations are densely clustered in a small region of the embedding space, making them nearly indistinguishable despite being annotated as different by human annotators. This phenomenon, known as representation collapse [19], indicates that prompt-based encoders alone struggle to capture fine-grained relational semantics. Consequently, such models may fail to generalize reliably to unseen or nuanced relations—a key limitation our approach aims to address.

To mitigate these issues, in this paper, we propose a prompt-contrastive learning framework specifically designed for zero-shot relation extraction, namely **PCRE**. Our framework addresses the aforementioned challenges through three tightly integrated components, each targeting a specific aspect of representation learning in the zero-shot setting.

First, to tackle the first limitation—the insufficient semantic modeling of relations under conventional prompt-based approaches—we go beyond simple template augmentation and adopt a knowledge-enhanced prompt-tuning paradigm. Specifically, we construct the verbalizer (i.e., a function that converts relation classes into natural language words or phrases) using a set of learnable virtual tokens to represent relations, thereby eliminating the need for manual prompt engineering [21]. Moreover, to fully exploit the rich semantics encoded in natural-language relation descriptions, we initialize the embedding of each answer (i.e., virtual token) with the average of the word embeddings from its corresponding relation description. These initialized embeddings are then fine-tuned end-to-end during prompt-tuning, allowing the model to absorb descriptive knowledge directly into the relation representations.

Second, to address the second limitation—the tendency of instance embeddings to collapse into indistinguishable clusters—we introduce an instance-level self-contrastive learning objective. Concretely, we generate two augmented views for each relation instance by concatenating it with two distinct prompt templates. The prompt-based encoder then produces two embeddings for the same underlying instance. During training, we minimize the distance between these two views while maximizing their separation from views of other instances. This contrastive mechanism effectively reshapes the embedding distribution, promoting diversity and preventing representation collapse, as illustrated in Figure 1b.

Third, to further enhance the alignment between instances and their semantic meanings, we propose an instance-description contrastive objective. In this component, the same prompt-based encoder is employed to generate embeddings not only for relation instances but also for all textual relation descriptions, creating a unified semantic space. The objective explicitly pulls each instance embedding closer to the embedding of its target relation’s description while pushing it away from embeddings of all other relation descriptions. This encourages the model to learn more compact and class-coherent representations for seen relations, which in turn improves generalization to unseen ones.

During the training stage, we jointly optimize the prompt-tuning loss together with both contrastive objectives—the instance-level self-contrastive loss and the instance-description contrastive loss—in an end-to-end manner. At inference time, we encode all test samples using the well-trained prompt encoder and apply the K-Means clustering algorithm over the resulting embedding space to perform zero-shot predictions for unseen relation types. To guide our investigation, we formulate the following research questions (RQs):

- **RQ1:** Does our proposed **PCRE** method perform better than competing methods?
- **RQ2:** Is each newly designed objective useful for the overall performance of **PCRE**?
- **RQ3:** Is the proposed **PCRE** robust to different numbers of training instances?
- **RQ4:** Can **PCRE** generate more compact instance representations for classification?

In terms of contribution, this paper investigates the task of zero-shot relation extraction (ZSRE) and makes at least three significant contributions:

- We propose a joint optimization approach that incorporates both prompt-tuning and instance-level self-contrastive learning into the prompt-based encoder. These two optimizations effectively leverage the knowledge in the pre-trained language model and address the problem of representation collapse.
- We introduce an instance-description contrastive learning approach that brings instances closer to their corresponding relation descriptions while separating them from others. This contrastive objective enables us to obtain more compact representations for each relation, enhancing their separability.
- To validate the superiority of **PCRE** in ZSRE, we conduct extensive experiments on two widely used relation extraction methods. The experimental results confirm the effectiveness of **PCRE** throughout the comparison with previous ZSRE  models.

The rest of the paper is organized as follows: We give an overview of related work in Section 2. Section 3 formally defines the task of ZSRE and introduces the workflow of our proposed **PCRE**. And then, Section 4 presents our proposed framework to perform zero-shot relation extraction, followed by experimental validation in Section 5. Section 6 concludes the paper.

## 2. Related Work

In this section, we review closely related work from three perspectives, including zero-shot relation extraction, prompt learning, and contrastive learning.

### 2.1. Zero-Shot Relation Extraction

Relation extraction (RE) is defined as determining the relation between two annotated entities given a piece of a sentence. To eliminate the cost of annotating training instances for fresh relations, recent research efforts have resorted to exploring zero-shot relation extraction. Since there are no training instances for test relations, existing methods are dependent on annotating auxiliary information for input and converting RE as different tasks [13]. As a pioneer work, Levy et al. reformulate RE as a reading comprehension task [14]. They design a question template for each relation and train a reading comprehension model which can infer whether the given sentence satisfies a relation. Differently, Obamuyide et al. define RE as a sentence entailment task [13]. They define relation descriptions for all relations, and a sentence entailment model is trained to predict whether an input sentence matches the description of a given relation. By converting RE into other tasks, semantic knowledge can be generalized from seen relations to unseen relations. Nevertheless, the mismatch between the surrogate task (e.g., question answering or textual entailment) and the intrinsic goal of relation classification often leads to imprecise semantic alignment, limiting zero-shot prediction accuracy—particularly for fine-grained or semantically similar relations.

To make use of knowledge in pre-trained BERT model directly, Chen et al. propose a ZS-BERT model to handle zero-shot relation extraction via attribute representation learning [15]. The representations for instances and relation descriptions can be derived from the BERT model, and ZS-BERT learns to match instance representations with description representations. The prediction of unseen relations for a test sample can be generated by searching its nearest relation description. Subjected to the advance of prompt learning, Xu et al. [17] design multiple prompt templates for an instance and derive representations with the assistance of prompts. Multiple representations of an instance are then fused via an attention mechanism and matched with the representations of relation descriptions. However, Xu et al. still fail to bridge the gap between the pre-training task of BERT and the task of relation extraction [17].

### 2.2. Prompt Learning

The prompt-tuning paradigm aims at bridging the gap between the pre-training and fine-tuning of pre-trained language models by formulating downstream tasks as a cloze-style (i.e., a fill-in-the-blank template such as “… is [MASK]”) task. Prompt learning was fueled by the birth of GPT [22] and has become popular across a wide range of natural language processing tasks. In the research community of information extraction, Hu et al. incorporate external knowledge into the prompt verbalizer for text classification [23]. Ding et al. introduce the idea of prompt learning into entity typing by constructing an entity-oriented verbalizer and templates [24]. To reduce reliance on labor-intensive prompt engineering, some works explore automatically generating answer words and templates from large language models, or searching for effective prompts via gradient-guided methods [16,25,26].

Prompt learning has also been investigated in relation extraction, achieving significant improvements over conventional approaches. Han et al. propose PTR (Prompt Tuning with Rules), which decomposes prompts using logical rules [27]. KnowPrompt injects entity and relation knowledge into PLMs to enhance semantic alignment [21]. However, these prompt-based RE methods are designed for supervised or few-shot settings, and often assume access to hand-crafted verbalizers or relation descriptions—resources that are unavailable in zero-shot scenarios.

This limitation highlights a key challenge: how to obtain high-quality semantic signals (e.g., relation descriptions) without human annotation. Recent advances in human–AI collaboration offer promising directions. For instance, ref. [28] shows that language models can learn to follow instructions through reinforcement learning from human feedback (RLHF), reducing dependence on expert-designed prompts. Similarly, ref. [29] leverages crowd-sourced feedback to guide model behavior in code generation, demonstrating the scalability of AI-augmented human input. Inspired by these works, we envision two practical extensions for zero-shot relation extraction: (1) AI-augmented relation descriptions: large language models can generate rich, context-aware descriptions for each relation (e.g., from relation names or seed instances), which could directly enhance our instance-description contrastive learning; (2) semantic labeling of clustered samples: for relations discovered via unsupervised clustering (e.g., unseen relations), LLMs can synthesize plausible descriptions from cluster prototypes, enabling contrastive training even without human labels. While our current framework assumes given descriptions, integrating such AI-generated semantic resources represents a natural path toward scalable, annotation-efficient zero-shot RE.

### 2.3. Contrastive Learning

In the field of computer vision, contrastive learning has attracted a lot of attention [30,31]. The idea behind contrastive learning is to pull data points from the same class together and push non-neighbor data points away. Intrinsically, contrastive learning enables instance representations from the neural model to be compact and better separated. The success of contrastive learning in computer vision tasks has aroused the interest to introduce it into the natural language processing field.

Recently, contrastive learning has mainly employed pre-trained language models in the field of natural language processing. Fang et al. augment data with a back-translation strategy and train a momentum encoder via contrastive learning [32]. Zhang et al. propose IS-BERT by adding an additional convolutional neural network layer on top of BERT and maximize the agreement between the global sentence representation and the corresponding local contextual representation [33]. Inspired by SimCLR [31], Gao et al. propose a framework named SimCSE for sentence representation learning where positive contrastive pairs are constructed with the use of two independently sampled dropout masks [34]. As a more comprehensive study, ConSERT investigates different data augmentation strategies, including adversarial attack, token shuffling, cutoff, and dropout for learning effective sentence representations by contrastive learning [19].

In summary, while existing zero-shot relation extraction methods either reformulate the task into reading comprehension or entailment [13,14], or rely on matching instance and description representations learned from BERT [15,17], they often overlook the structural alignment between instances and semantic descriptions during representation learning. Prompt-based approaches [21,27] have demonstrated strong performance in supervised settings but are rarely adapted to zero-shot scenarios, and most do not leverage relation descriptions to initialize or guide prompt representations. Meanwhile, contrastive learning techniques in NLP [19,34] typically focus on sentence-level augmentation (e.g., via dropout or token shuffling) and lack explicit modeling of class semantics—especially critical in zero-shot tasks where labels are defined only by textual descriptions.

In contrast, our proposed PCRE framework uniquely integrates three key ideas: (1) it adapts prompt learning to the zero-shot relation extraction setting; (2) it initializes prompt-based relation representations using natural-language descriptions, thereby grounding them in semantic knowledge; and (3) it introduces a dual contrastive mechanism—combining instance-level self-augmentation (via multiple prompts) and instance-description alignment—to learn compact, separable, and semantically coherent embeddings. This holistic design enables PCRE to bridge the gap between pre-training objectives, prompt formulation, and zero-shot generalization in a way that prior methods do not.

## 3. Preliminary

In this section, we give a formal definition for the task of zero-shot relation extraction (ZSRE), and then introduce the workflow of our proposed framework **PCRE**.

### 3.1. Task Definition

Relation extraction is formally defined as identifying the semantic relation r∈R between a pair of entities $\left(e_{h},e_{t}\right)$ according to the clues in a piece of sentence *x* mentioning the entity pair. The notation $x=\{w_{1},w_{2},\cdots ,w_{L}\}$ denotes the word sequence of the instance, where $w_{i}$ is *i*-th token in the sequence and *L* is the sequence length. $e_{h}$ and $e_{t}$ represent the head and tail entity mentions, and R is the relation set.

Under the setting of zero-shot relation extraction (ZSRE), there are two sets of relations, a set of seen relations (i.e., those observed during training) $R_{s}=\left\{r_{s}^{1},\cdots ,r_{s}^{n}\right\}$ for training and a set of unseen relations (i.e., not present in training data) $R_{u}=\left\{r_{u}^{1},\cdots ,r_{u}^{m}\right\}$ for test. The training set $D={\left\{s_{i}=\left(x_{i},e_{ih},e_{it},r_{i}\right)\right\}}_{i=1}^{N}$ consists of *N* annotated relational instances whose relations are pre-defined in $R_{s}$. ZSRE  requires us to train a relation extraction model M based on the training data, i.e., $M\left(s_{i}\right)\to r_{i}\in R_{s}$. After training, given a test sample $s_{i}^{\prime }=\left(x_{i}^{\prime },e_{ih}^{\prime },e_{it}^{\prime }\right)$, the well-trained relation extraction model M can be harnessed to predict the unseen relation $r_{i}^{u}$, i.e., $M\left(s_{i}^{\prime }\right)\to r_{i}^{u}\in R_{u}$.

### 3.2. Overview

To handle relation extraction in the zero-shot setting, we propose a novel framework, namely **PCRE**, based on prompt-contrastive learning. Our goal is to learn transferable knowledge from training data about how to separate seen relations and generalize this knowledge to unseen relations at test time.

**Figure 2:**
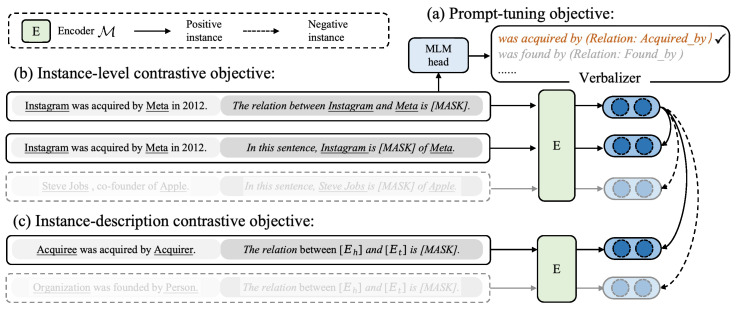
The framework of **PCRE** which is optimized with three objectives, including prompt-tuning objective, instance-level contrastive objective, and instance-description contrastive objective. The detailed description of each component and the three training objectives are provided in Section 3.2.

As depicted in Figure 2, the workflow of **PCRE** involves training the model M with three complementary objectives to jointly capture semantic meaning and geometric structure in the embedding space. For each relation instance, we concatenate it with two distinct prompt templates to construct two augmented views (each prompt template contains a [MASK] position to be filled with a relation-specific label word). These views serve as inputs to a shared prompt-based encoder.

The first objective follows the prompt-tuning paradigm. Instead of using handcrafted verbalizers, we map each seen relation to a learnable virtual token. Crucially, the embedding of each virtual token is initialized with the average of the word embeddings from its corresponding natural-language relation description and is updated during training. This allows the model to ground relation representations in explicit semantic knowledge, enabling better generalization to unseen relations.

To address the issue of representation collapse—where diverse instances are mapped to indistinguishable embeddings—we introduce an instance-level contrastive learning objective. Specifically, the two augmented views generated from the same instance are treated as a positive pair, while views from other instances serve as negative samples. The contrastive loss pulls the positive pair closer in the embedding space and pushes them away from all negatives, thereby encouraging intra-relation consistency and inter-relation separation.

Furthermore, to ensure that instance representations are semantically aligned with their target relations, we design an instance-description contrastive learning objective. Using the same prompt-based encoder, we also embed all textual relation descriptions into the same space. For each instance, this objective minimizes the distance between its embedding and the embedding of its correct relation description while maximizing distances to all other description embeddings. This yields more compact and class-coherent clusters for seen relations, which facilitates clustering-based prediction for unseen ones.

By jointly optimizing M with these three objectives, our framework learns rich, structured, and semantics-aware representations that effectively separate relations in the zero-shot scenario.

At the test phase, we input all test samples into the trained prompt-based encoder and cluster the resulting instance embeddings into m groups using K-Means. To assign relation labels in a zero-shot manner, we associate each cluster centroid with the unseen relation whose description embedding is closest in cosine similarity.

## 4. Methodology

This section introduces our proposed **PCRE** model to accomplish zero-shot relation extraction. Sequentially, we describe our prompt-based encoder in Section 4.1, model training objectives in Section 4.2, and relation inference module in Section 4.3.

### 4.1. Prompt-Based Encoder

The prompt-based sentence encoder aims to generate the informative contextual representation of each relational instance with the assistance of a prompt. In this paper, we employ a pre-trained BERT  model [35] as our relation extraction model (other pre-trained language models can also be harnessed as the backbone encoder), denoted as M. Generally, before inputting an instance into M, we need to convert the instance *s* into a token sequence. We add four additional entity markers surrounding two entities to allow M to be aware of the entity positions:

(1) $$\begin{array}{cc} \hat{s}=\{ & \left[\mathrm{CLS}\right],w_{1},\cdots ,\left[E_{h}\right],w_{i},\cdots ,w_{j},\left[/E_{h}\right],\cdots ,\\ \; & \left[E_{t}\right],w_{k},\cdots ,w_{l},\left[/E_{t}\right],\cdots ,w_{L},\left[\mathrm{SEP}\right]\}. \end{array}$$

In the token sequence, [CLS] and [SEP] mark the beginning and the end of the instance. $\left[E_{h}\right]$, $\left[/E_{h}\right]$, $\left[E_{t}\right]$, and $\left[/E_{t}\right]$ mark the beginning and the end of the head and tail entity mentions. Afterwards, M maps $\hat{s}$ into a sequence of hidden vectors $\left\{h_{k}\in R^{d}\right\}$, where *d* is the hidden dimension of the language model.

In the traditional fine-tuning paradigm for relation extraction [11], the hidden vectors corresponding to the $\left[E_{h}\right]$ and $\left[E_{t}\right]$ positions are concatenated to derive a contextualized relation representation $h\in R^{2d}$:

(2) $$h=h_{\left[E_{h}\right]}⊕h_{\left[E_{t}\right]}.$$

For classifying relations, a task-specific head should be added and optimized, i.e.,

(3) $$\mathrm{softmax}(W_{o}r+b_{o}),$$

where $W_{o}\in R^{n\times 2d}$ and $b_{o}\in R^{n}$ are learnable parameters for classification. Despite the success of fine-tuning a pre-trained BERT model, some recent studies found the significant gap of objective forms in masked language pre-training and the aforementioned fine-tuning restricts taking full advantage of knowledge in pre-trained language models [27].

An alternative approach to solving the problem is prompt tuning, where we need to formulate the relation extraction as a cloze-style masked language prediction task [21,27]. To achieve this goal, we augment data samples with an appropriate template T(·,·) to prompt the instance *s*. Except for retaining its original tokens, a [MASK] token is necessarily held in the prompt template. In our framework, we equip *s* with a template and generate the prompt input as:

(4) $$\tilde{s}=“\left[\mathrm{CLS}\right]\;\hat{s}\;\left[\mathrm{SEP}\right]\;T_{i}\left(e_{h},e_{t}\right)\;\left[\mathrm{SEP}\right]”,$$

where $e_{h}$ and $e_{t}$ represent the head and tail entity mentions. To be specific, we construct two templates:

(5) $$\begin{array}{cc} T_{1}\left(e_{h},e_{t}\right) & =“\mathrm{The}\;\mathrm{relation}\;\mathrm{between}\;e_{h}\;\mathrm{and}\;e_{t}\;\mathrm{is}\;\left[\mathrm{MASK}\right]”,\\ T_{2}\left(e_{h},e_{t}\right) & =“\mathrm{In}\;\mathrm{the}\;\mathrm{sentence},\;e_{h}\;\mathrm{is}\;\left[\mathrm{MASK}\right]\;\mathrm{to}\;e_{t}”. \end{array}$$

By inputting the prompt-augmented instance into the model M, we can derive a sequence of hidden vectors. The hidden vector $h_{\left[\mathrm{MASK}\right]}$ of the [MASK] position is taken as the representation of an instance.

In addition, we use the same method to encode relation descriptions. For relation *r*, we augment the description *d* with the aforementioned prompt templates as Equation (4), but the difference is that we replace the entity mentions in the template with entity markers, i.e.,:

(6) $$\tilde{d}=“\left[\mathrm{CLS}\right]\;d\;\left[\mathrm{SEP}\right]\;T_{i}\left(\left[E_{h}\right],\left[E_{t}\right]\right)\;\left[\mathrm{SEP}\right]”.$$

We also use the hidden vector of the [MASK] position as the representation of a relation description.

### 4.2. Model Training Objectives

After obtaining the representations of instances and relation descriptions, we propose three objectives to jointly optimize the relation extraction model M.

#### 4.2.1. Prompt-Tuning Objective

Prompt tuning aims at enabling the model M to learn semantic features. To get rid of labor-intensive prompt engineering, we expand M with a set of learnable virtual words V to completely represent the corresponding seen relations $R_{s}$. In other words, instead of a regular verbalizer that maps one relation to one label word in the vocabulary, we assume that each virtual word $v_{i}\in V$ can describe the implicit semantics of relation $r_{i}\in R_{s}$. To leverage the semantic information in relation $r_{i}$, we initialize the embedding of $v_{i}$ with the average embedding of words in corresponding relation description $d_{i}$.

Afterwards, the model M is tasked with predicting which word is appropriate to fill in the [MASK] position for relation extraction. In this way, we can employ M to generate the hidden vector $h_{\left[\mathrm{MASK}\right]}$ of the [MASK] position, and then model the probability of predicting relation $r_{i}\in R_{s}$:

(7) $$p\left(r_{i}|h_{\left[\mathrm{MASK}\right]}\right)=\frac{\exp(v_{i}\cdot h_{\left[\mathrm{MASK}\right]})}{\sum_{r_{j}\in R_{s}}\exp\left(v_{j}\cdot h_{\left[\mathrm{MASK}\right]}\right)},$$

where exp(·) denotes the exponential function ($\exp\left(z\right)=e^{z}$).

With access to the training set $D={\left\{s_{i}=\left(x_{i},e_{ih},e_{it},r_{i}\right)\right\}}_{i=1}^{N}$, the model M can be prompt-tuned by minimizing the cross-entropy loss:

(8) $$L_{CE}=-\log p\left(r_{i}|h_{\left[\mathrm{MASK}\right]}\right).$$

#### 4.2.2. Instance-Level Contrastive Objective

To reshape the feature space of the pre-trained BERT model, we design an instance-level contrastive learning objective to pull two views of an instance together and push apart from other instances. Furthermore, this objective can maximize inter-relation distance and minimize intra-relation variance [36], such that the model M can better find subtle differences between relations and separate easily confused relation pairs.

In this module, we adopt the temperature-scaled cross-entropy loss as the contrastive objective. Given a piece of an instance, we derive two representations from the two augmented views as $h_{1}=\mathrm{Proj}\left(h_{1[\mathrm{MASK}]}\right)$ and $h_{2}=\mathrm{Proj}\left(h_{2[\mathrm{MASK}]}\right)$, where $h_{i[\mathrm{MASK}]}$ is the hidden vector of the [MASK] position in the *i*-th template. The function Proj(·) consists of two feed-forward layers and a GeLU activation function. Therefore, for a mini-batch with *K* samples, we can obtain 2K representations, denoted as $H=\{h_{1},\cdots ,h_{2K}\}$. Each representation is trained to find out its counterpart view of the same instance from all other 2K−1 in-batch representations:

(9) $$L_{ICL}=-\log\frac{\exp(\mathrm{sim}\left(h_{i},h_{j}\right)/\tau )}{\sum_{k=1}^{2K}1_{\left[i\neq k\right]}\exp\left(\mathrm{sim}\left(h_{i},h_{k}\right)/\tau \right)},$$

where τ is an adjustable temperature hyper-parameter, $1_{\left[\cdot \right]}$ is an indicator function, and $\mathrm{sim}(h_{i},h_{j})$ is the cosine similarity function which is defined as $\frac{h_{i}^{⊤}h_{j}}{∥h_{i}∥\cdot ∥h_{j}∥}$.

#### 4.2.3. Instance-Description Contrastive Objective

Except for instance-level contrastive learning, we propose a novel instance-description contrastive loss to learn more compact representations for each relation. Since a relation description defines the semantic concept of a relation, for a given relation, we treat its description representation as an anchor in the feature space and encourage all corresponding instance embeddings to cluster around it.

Crucially, to explicitly separate different relations, our loss function actively enlarges the distances between an instance embedding and the embeddings of negative (i.e., non-matching) relation descriptions. This is achieved through the structure of the contrastive objective: during training, every relation description other than the correct one is treated as a negative sample. As the model minimizes the loss, it not only pulls the instance closer to its target description but also pushes it away from all incorrect descriptions. Because the denominator in the loss aggregates similarities to all relation descriptions—including negatives—this repulsive effect directly increases the geometric distances between the instance and irrelevant relation anchors, thereby sharpening the decision boundaries between relation clusters.

To achieve this goal, we also define the instance-description contrastive objective as a temperature-scaled cross-entropy loss. For each relation, we input the prompt-augmented relation description into the prompt-based encoder and derive the hidden vector of the [MASK] position. Let $R=\{r_{1},\cdots ,r_{n}\}$ denote the embeddings of relation descriptions. For a mini-batch of samples, each data point is trained by pulling close to its target relation representation and pushing apart from negative relation representations.

(10) $$L_{DCL}=-\log\frac{\exp(\mathrm{sim}\left(h_{i},r_{j}\right)/\tau )}{\sum_{k=1}^{n}\exp\left(\mathrm{sim}\left(h_{i},r_{k}\right)/\tau \right)}.$$

Finally, to learn both seen relational semantics and compact instance embeddings, we jointly optimize ZSRE model M with the cross-entropy loss and two contrastive losses in a weighted manner:

(11) $$L_{new}=\lambda_{1}\cdot L_{CE}+\lambda_{2}\cdot L_{ICL}+\lambda_{3}\cdot L_{DCL},$$

where $\lambda_{1}$, $\lambda_{2}$, and $\lambda_{3}$ are three weights for different constitute losses.

### 4.3. Relation Inference Module

At the phase of testing, we send incoming instances of the unseen relation into the well-learned model M to generate their representations. We apply the K-Means algorithm to partition the representations into *m* clusters, where *m* is the number of unseen relations defined in Section 3. To align each cluster with relation labels, we calculate the distances between cluster centroids and relation description representations. For all clusters, we can derive the cluster centroids by averaging all instance embeddings, and the centroid set is denoted as $C=\{c_{1},\cdots ,c_{m}\}$. Moreover, we harness the prompt-based encoder to generate description representations for all predicted relations as $R^{\prime }=\left\{r_{1}^{\prime },\cdots ,r_{m}^{\prime }\right\}$.

We can then calculate the probabilities of the unseen relations in $R_{u}$ for each cluster as follows,

(12) $$p\left(r_{u}^{i}|c_{j}\right)=\frac{\exp(-d\left(c_{j},r_{i}^{\prime }\right))}{\sum_{k=1}^{m}\exp\left(-d\left(c_{j},r_{k}^{\prime }\right)\right)},$$

where d(·,·) is the distance function between two vectors. There are multiple choices for the distance function. In this paper, we adopt Euclidean distance for our proposed method **PCRE** due to its superior performance.

## 5. Experiments

In this section, we first introduce the experimental setup, and then conduct extensive experiments to answer the research questions in Section 1.

### 5.1. Experimental Setup

#### 5.1.1. Datasets

We conduct our experiments on two widely used relation extraction datasets:

FewRel consists of relations primarily from Wikipedia biographies, covering person-related facts such as place of birth, employer, spouse, and education, containing 80 relations with 700 annotated instances per relation. To support zero-shot relation extraction, we randomly select parts of relations as seen relations for training and the rest of the relations as unseen relations for testing. To be specific, we set the number of unseen relations *m* to 15, 20, 30, and 40.

TACRED is derived from news articles and includes a broader range of relations, including both person-centric and organization-centric relations, containing 42 relations, and each relation therein has different quantities of instances. To reduce the impact of data imbalance on test performance, we limit the number of instances of each relation to 1000. Similarly, we test all models with different unseen relations, where we set *m* to 11, 15, and 20.

As well as training ZSRE models with full training instances, we also design few-shot settings to validate the robustness of our **PCRE**. For each seen relation, we randomly select 20, 100, and 200 pieces of instances for training to evaluate **PCRE** in few-shot scenarios.

#### 5.1.2. Experimental Settings

In the implementation of our proposal, we adopt the base version of BERT [35] as the backbone encoder and set the learning rate for it to 1 × 10^−5^ with an Adam optimizer [37]. The adjustable temperature for contrastive objectives is selected as 0.05 across two datasets. The weights for different losses $\lambda_{1}$, $\lambda_{2}$, and $\lambda_{3}$ are set to 0.8, 0.1, and 0.1, respectively. Before evaluating all ZSRE models, we train them for 2 epochs. During training, each mini-batch contains 64 pieces of instances and each instance is restricted to 200 word tokens.

Following previous work [15], we adapt the standard F1 score as the evaluation metric. To comprehensively compare the performance of baselines and our proposal, we also use the NMI indicator to measure the shared information between the predicted relations and the ground truths. Moreover, a similar metric ARI is harnessed to measure the agreement degree between the cluster and golden distribution. The more performant the model, the higher these indicators. We run all experiments five times and report the average indicator values.

#### 5.1.3. Competing Methods

To show the superiority of **PCRE** when performing zero-shot relation extraction, we compare it with a variety of baselines. Among the baselines, some methods convert ZSRE into other task formulations, including a text entailment-based model **ESIM**  [13] and a reading comprehension-based model **QARE**  [14]. Two representation-based methods **ZS-BERT**  [15] and **MTB**  [38] are employed for comparison, which predict the relation by searching the closest description to an instance. Additionally, we employ prompt-based approaches as our competing methods, which can activate the internal knowledge in pre-trained language models for zero-shot relation extraction, including **RelationPrompt**  [39] and **MultiPrompt**  [17]. For fair comparison, we harness the base version of BERT as the backbone encoder for all methods.

Table 1 and Table 2 answer **RQ1** by showing the results of our model against competing methods on two real-world datasets. We vary the number of unseen relations in [15,20,30,40] for FewRel and [10,15,20] for TACRED. In summary, our proposed **PCRE** significantly outperforms competing methods on these two datasets.

### 5.2. Overall Performance

**RQ1:** *Does our proposed **PCRE** method perform better than competing methods*?

From the results, we have the following observations: (1) On two datasets, **PCRE** obtains better performance than all baseline methods under all settings. Since the joint optimization objective of **PCRE** targets modeling semantic and spatial features, we can obtain compact representations of instances, which benefits the robust performance of **PCRE** across different numbers of unseen relations. (2) The two baselines which define ZSRE as other task formulations, **ESIM** and **QARE**, generate unsatisfactory performance due to the divergence in task formulations. **ZS-BERT** and **MTB** generate instance representations without taking the prompt as input, resulting in them failing to make full use of the knowledge in pre-trained language models. (3) The strongest baseline is **MultiPrompt** which harnesses multiple prompt templates to assist high-quality instance representations. However, its standard fine-tuning method rather than prompt tuning gives rise to its inferior performance in comparison with **PCRE**.

### 5.3. Ablation Study

**RQ2:** *Is each newly designed objective useful for the overall performance of **PCRE***?

**Figure 3:**
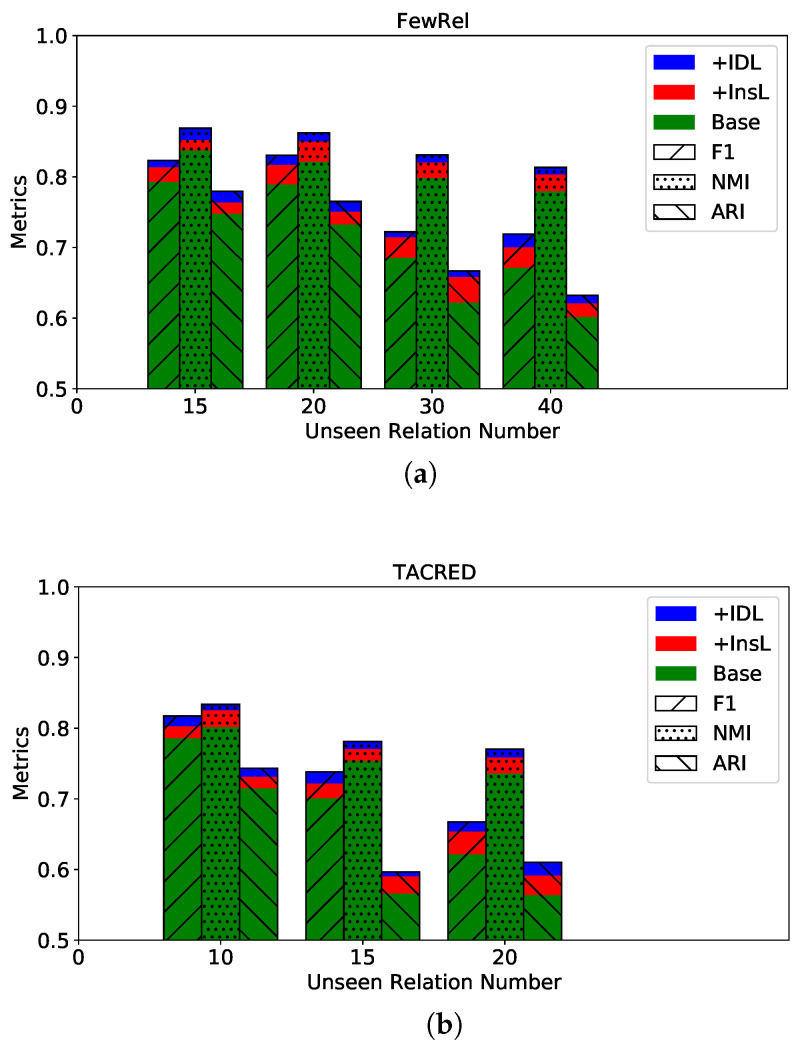
Ablation study on two dataset, (**a**) for FewRel and (**b**) for TACRED.

To answer **RQ2**, we conduct ablation studies on two datasets by adding a proposed objective each time. Specifically, we first construct a base version of **PCRE** by only maintaining prompt-tuning loss (the bar “base” in Figure 3). And then, we add the instance-level contrastive objective (the bar “+InsL” in Figure 3) and instance-description contrastive objective (the bar “+IDL” in Figure 3) sequentially.

It reads from the results that: (1) With the sequential addition of each proposed objective, the performance of our model in ZSRE is gradually promoted. The observation indicates that each proposed objective contributes to the overall performance of **PCRE**. (2) The instance-level contrastive objective brings a larger improvement to **PCRE** than the instance-description contrastive objective. It implies that the collapse problem does harm to ZSRE which can be mitigated by the instance-level contrastive objective. Therefore, we can safely conclude that each newly designed contrastive objective plays a key role in generating discriminative instance representations.

### 5.4. Few-Shot Analysis

**RQ3:** *Is the proposed* ***PCRE*** *robust to different numbers of training instances?*

**Figure 4:**
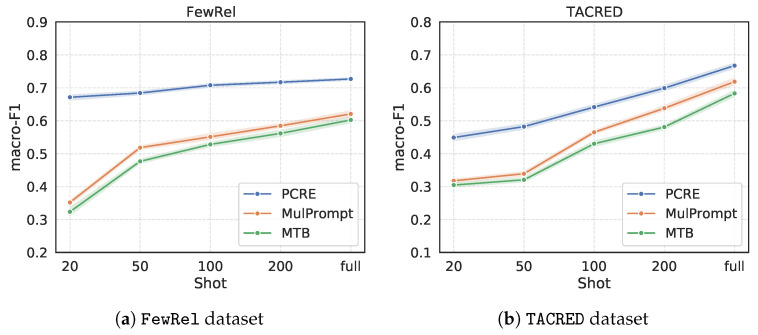
Analysis of few-shot scenarios on two datasets, (**a**) for FewRel and (**b**) for TACRED.

This set of experiments is designed to answer **RQ3** by varying the number of training instances of each relation in [20, 50, 100, 200, full] shots. To be clear, Figure 4 only compares the F1 scores of **PCRE** with the two strongest competing methods among baselines, **MTB** and **MultiPrompt**, where we set the number of unseen relations to 40 and 20 for the FewRel and TACRED datasets, respectively.

From the fluctuation of the plots in Figure 4, we can see that our **PCRE** achieves robust performance across different few-shot settings. To be specific, using 20 pieces of instances of each training relation only leads to less than 4% F1 score degradation. However, the F1 scores of two baseline methods drop dramatically with the decrease in training samples. For **PCRE**, over 100 pieces of training instances, the performance growth will slow down. When the number of instances reaches 200, the performance of **PCRE** is close to that using full training shots. It proves that our proposed **PCRE** has better learning efficiency for fewer training samples. The first reason is that **PCRE** is based on the prompt-learning methods which are effective for few-shot learning. Additionally, the second reason is that **PCRE** benefits from two contrastive objectives which are helpful to generate discriminative instance representations.

### 5.5. Embedding Visualization

**RQ4:** *Can* ***PCRE*** *generate more compact instance representations for classification?*

**Figure 5:**
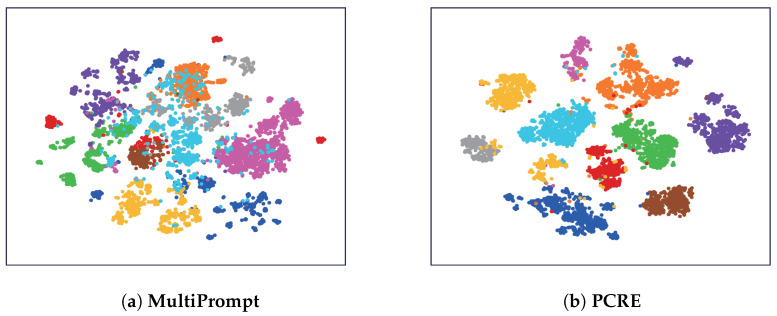
t-SNE visualization of 10 unseen relation representations derived by **MultiPrompt** and **PCRE** on FewRel dataset.

To address **RQ4**, we utilize t-SNE [20] to intuitively show the representations of unseen relations by mapping them into 2D data points. We conjecture that the outstanding performance of **PCRE** in zero-shot relation extraction benefits from deriving compact instance representations which are easier to separate. In this set of experiments, we select **MultiPrompt** as our competing method and generate instance representations of 10 unseen relations. In Figure 5, each data point is colored according to its golden relation label.

As we observe from Figure 5a, the data points generated by **MultiPrompt** mingle among different clusters, especially for the blue points. The observation indicates that **MultiPrompt** fails to distinguish similar relations well although it harnesses prompts to generate high-quality instance representations. On the contrary, our proposed **PCRE** is optimized with two additional contrastive objectives and derives more discriminative instance representations as shown in Figure 5b. The results demonstrate the effectiveness of **PCRE** in dealing with similar relations. With the assistance of two additional objectives, our approach can learn the difference between instances and the difference between relations.

## 6. Conclusions

In this paper, we propose a prompt-contrastive learning framework for zero-shot relation extraction, namely **PCRE**. The proposed solution addresses the shortcomings of current zero-shot relation extraction models in learning effective representations from the pre-trained BERT model. Therefore, our proposed model is more robust in dealing with data in a wider range of domains involving relational knowledge acquisition.

To mask the full use of semantic knowledge behind the relations and transfer to predicting unseen relations, we first adopt prompt tuning to optimize our model with a soft verbalizer, which are initialized by relation descriptions and updated during training. In addition, confronted with the feature collapse problem, we propose an instance-level contrastive objective with the assistance of multi-prompt learning. Furthermore, we devise a novel instance-description contrastive objective to learn more compact representations for instances. With a joint optimization, the proposed **PCRE** model can learn discriminative embeddings for better separating relations. Extensive experiments can safely validate the superiority of our proposal over previous methods.

As for future work, we are interested in investigating active learning for zero-shot relation extraction. By annotating a handful of key instances, the performance of zero-shot relation extraction models when predicting novel relations can be dramatically improved.

## Figures and Tables

**Table 1:** Experimental results (%) produced by baselines and our proposal on the FewRel dataset in terms of the standard F1 score, NMI, and ARI. *m* is the number of unseen relations for testing, and we vary *m* in [15, 20, 30, 40] to observe the performance changes.

Methods	*m* = 15			*m* = 20			*m* = 30			*m* = 40		
	F1	NMI	ARI	F1	NMI	ARI	F1	NMI	ARI	F1	NMI	ARI
**ESIM**	51.89	61.78	42.75	35.23	40.86	27.11	28.25	47.13	21.52	26.65	51.62	21.84
**QARE**	55.40	62.53	50.78	47.98	55.31	42.77	40.79	55.26	36.87	36.49	55.64	33.73
**ZS-BERT**	63.33	70.70	59.24	55.78	62.90	51.44	46.43	61.66	42.94	45.68	64.43	42.68
**MTB**	78.62	83.57	74.83	72.38	78.24	68.54	63.51	76.61	59.98	60.35	75.90	54.54
**RelationPrompt**	71.45	76.34	67.29	70.32	75.55	65.40	60.41	65.46	56.72	56.33	62.10	51.42
**MultiPrompt**	76.88	81.47	72.88	73.49	78.96	69.32	63.89	77.21	61.04	61.97	76.92	55.65
**PCRE**	**82.33**	**86.89**	**77.96**	**83.04**	**86.25**	**76.52**	**72.21**	**83.13**	**66.67**	**71.91**	**81.33**	**63.25**

**Table 2:** Experimental results (%) produced by baselines and our proposal on the TACRED dataset in terms of the standard F1 score, NMI, and ARI. We vary the number of unseen relations *m* in [10,15,20].

Methods	*m* = 10			*m* = 15			*m* = 20		
	F1	NMI	ARI	F1	NMI	ARI	F1	NMI	ARI
**ESIM**	52.44	61.70	41.87	47.16	53.32	39.65	38.48	46.53	31.56
**QARE**	56.53	64.22	47.35	52.12	58.66	45.96	43.22	54.45	37.42
**ZS-BERT**	65.44	70.54	54.79	61.25	67.34	52.11	51.75	60.04	45.53
**MTB**	76.43	81.52	72.45	66.47	72.04	62.15	58.78	65.12	52.08
**RelationPrompt**	73.37	78.66	69.52	64.35	68.22	57.64	55.43	62.76	49.54
**MultiPrompt**	78.79	82.42	73.45	68.46	72.19	59.45	61.85	67.35	56.32
**PCRE**	**81.71**	**83.36**	**74.31**	**73.83**	**78.12**	**64.63**	**66.72**	**77.03**	**61.01**

## Data Availability

The data presented in this study are available on request from the corresponding author.
